# Personalized prediction of optimal water intake in adult population by blended use of machine learning and clinical data

**DOI:** 10.1038/s41598-022-21869-y

**Published:** 2022-11-16

**Authors:** Alberto Dolci, Tiphaine Vanhaecke, Jiqiong Qiu, Riccardo Ceccato, Rosa Arboretti, Luigi Salmaso

**Affiliations:** 1 Health, Hydration and Nutrition Science Department, Danone Research, Route Départementale 128, 91767 Palaiseau, France; 2 Department of Management and Engineering, University of Padova, Vicenza, Italy; 3 Department of Civil, Environmental and Architectural Engineering, University of Padova, Padua, Italy

**Keywords:** Physiology, Kidney, Metabolism, Biomarkers, Nutrition, Machine learning, Scientific data

## Abstract

Growing evidence suggests that sustained concentrated urine contributes to chronic metabolic and kidney diseases. Recent results indicate that a daily urinary concentration of 500 mOsm/kg reflects optimal hydration. This study aims at providing personalized advice for daily water intake considering personal intrinsic (age, sex, height, weight) and extrinsic (food and fluid intakes) characteristics to achieve a target urine osmolality (U_Osm_) of 500 mOsm/kg using machine learning and optimization algorithms. Data from clinical trials on hydration (four randomized and three non-randomized trials) were analyzed. Several machine learning methods were tested to predict U_Osm_. The predictive performance of the developed algorithm was evaluated against current dietary guidelines. Features linked to urine production and fluid consumption were listed among the most important features with relative importance values ranging from 0.10 to 0.95. XGBoost appeared the most performing approach (Mean Absolute Error (MAE) = 124.99) to predict U_Osm_. The developed algorithm exhibited the highest overall correct classification rate (85.5%) versus that of dietary guidelines (77.8%). This machine learning application provides personalized advice for daily water intake to achieve optimal hydration and may be considered as a primary prevention tool to counteract the increased incidence of chronic metabolic and kidney diseases.

## Introduction

In recent years a sharp increase in the prevalence of metabolic and kidney diseases across the general population has been observed^1,2^. Growing evidence shows that body fluid imbalances, which result in elevated hydration biomarkers such as concentrated urine, contribute to negative health outcomes or life-threatening situations^3–7^. In particular, low water intake and concentrated urine have been adversely linked to chronic kidney disease progression^2,8–10^, kidney stones incidence^11^ and glucose dysregulation^12,13^. Additionally, interventional studies showed that increased fluid intake improved renal functions^14,15^ in the general population, and greatly decreased urinary tract infection (UTI) recurrence rate^16^ in adult women. Low water intake is also a global public health challenge as recent research assessing fluid intake habits across different countries worldwide highlights that about 50% of the study adult population and more than 50% of the child and adolescent study population did not comply with the European Food Safety Authority (EFSA) Adequate Intake of water from fluids^17,18^. Despite existing recommendations for water intake and scientific evidence on the role of water for health, today, a considerable portion of the population is at risk of hydration-related health consequences such as metabolic and kidney disease.

It was previously proposed that a threshold value of daily urinary concentration may identify optimal fluid balance and provide a target to aim for^19,20^. Several recent intervention studies, including randomized controlled trials, have now demonstrated that lowering 24 h urine osmolality (U_Osm_) to 500 mOsm/kg or below, can reduce a predictive marker of cardiovascular risk, namely arginine vasopressin as measured by copeptin^16,21–23^, as well as reduce UTI incidence^16^. Collectively, these experimental results suggest that optimal hydration may be achieved by drinking sufficient water to reach this daily target for urine concentration. While current government-issued dietary guidelines consider the average need of a population, evidence sheds light on the opportunity for implementing novel, individual-centric interventions to improve hydration among the general population^24^. In this context, personalized hydration approach aims to develop specific and comprehensive water advice. This methodology accounts for the physiological requirements of an individual^25,26^ based on phenotypic characteristics, analysis of current behavior, preferences, barriers, and objectives.

Physiological requirements for fluids are the result of a continuous and complex interplay between an individual’s intrinsic and extrinsic factors which challenge homeostasis. Consequently, fluid balance, as a key player in our ability to maintain homeostasis, should be regarded as one of the main components for the provision of a personalized hydration intervention. In general terms, factors that may affect fluid balance are sex, body mass composition^27^, physical activity, thermoregulatory processes^28^, and medical conditions^29^. As human beings constantly lose water through urine and insensible water losses, the only way to replenish total body water is to drink water from an external source. Considering the dynamic nature of the homeostatic processes that ultimately determine fluid balance, we considered machine learning (ML) statistical analysis, a relevant and novel approach, to reflect these dynamics. Datasets from multiple clinical studies on hydration were used as a valid source of data for this investigation. The aim was to provide personalized advice on daily water intake considering personal intrinsic and extrinsic variability to achieve a target 24 h U_Osm_ of 500 mOsm/kg in a subset of healthy adults excluding athletes and pregnant and lactating women.

## Subjects and methods

### Study population

**Figure 1:**
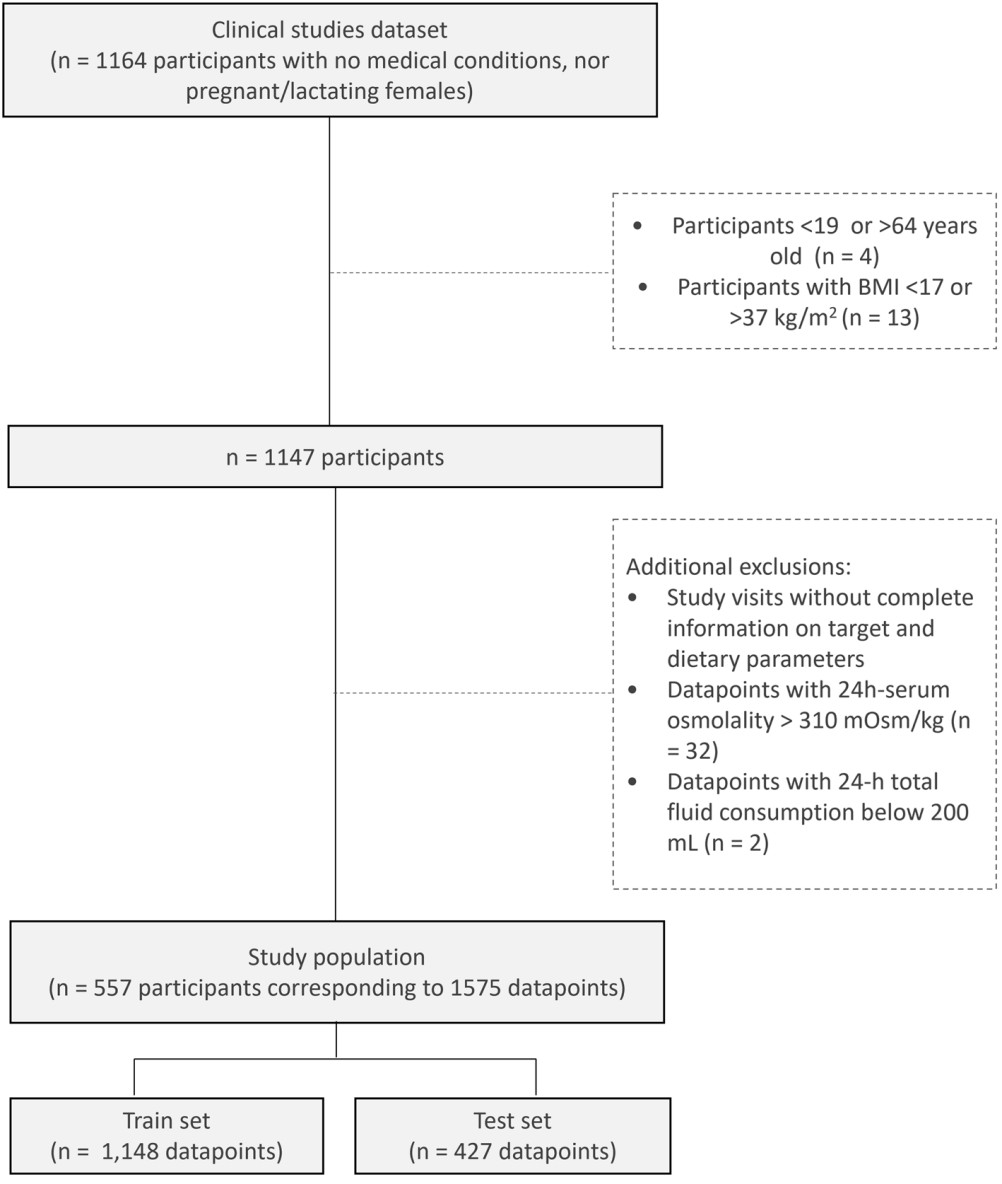
Participant flowchart.

All research was conducted according to the ethical principles stated in the Declaration of Helsinki. All subjects provided written informed consent. Each study was approved by a local Ethics Committee (Comité Etico De Investigacion Clinica (CEIC) del Hospital Universitario La Princesa, Madrid, Spain; CEIC Hospital Universitario de La Paz, Madrid, Spain; Comité de Ética e Investigación para Estudios en Humanos (CEIEH), Mexico, Mexico; Comité de Bioética Para la Investigacion Clinica, Mexico, Mexico; Comité de Protection des Personnes (CPP) of Ile de France XI, Paris, France; Ethics Committee CPP Sud Méditerranée III, Nîmes, France; Ethics Committee CPP Est IV, Strasbourg, France; Ethics Committee CPP Est III, Nancy, France; Ethics Committee of COMAC Medical, Sofia, Bulgaria; Supplemental Methods). Multiple datasets from previous clinical trials on hydration and fluid balance (4 open label randomized controlled trials, and 3 open label non randomized trials in parallel groups) were merged into one single dataset (*n* = 1164 participants) (Fig. 1). Each subject had 107 possible variables collected during the clinical studies at each single time point. Some participants had measurements of their biological variables performed on multiple timepoints during the same clinical study, consequently their information was arranged on multiple lines for homogeneity within the dataset. Participants not meeting the aforementioned criteria on age (*n* = 4) and BMI (*n* = 13) were filtered out. Datapoints from study visits without complete information on biological and dietary parameters were filtered out. Additionally, subjects' datapoints were further filtered out if Plasma Osmolality (P_Osm_) was above 310 mOsm/kg (*n* = 32), a threshold for dehydration which corresponds to a ~ 5% body weight loss, a condition not generally met by the general population^30^. Also, participants with a 24 h total fluid consumption below 200 mL (*n* = 2) were excluded on the basis that such fluid intake cannot meet fluid balance physiological requirements in general population. This resulted in a dataset of 1575 rows reporting information of 557 subjects. Within the resulting dataset, some participants had missing fluid intake data (*n* = 107). Nonetheless, to maintain a fair representation of the subject population and to not potentially undermine statistical analysis capability, these participants were retained in the dataset. This choice was driven by the capability that the ML approach has to deal with missing values, and it was worth considering the wealth of information these participants recorded for other variables. Following this process, participants were randomly split according to their participant ID number with a ratio of 75:25, ensuring that each participant was assigned to either the training set or the test set. The final dataset consisted in 1,148 rows for the train test and 427 rows for the test set.

### Feature selection

Extreme Gradient Boosting (XGBoost) was adopted to achieve the preliminary identification of the most relevant features linked to urine production in the dataset (Hyperparameters tuning in Supplemental Methods).

### Machine learning algorithms

Once the most relevant features were identified in the dataset, several ML methods were used to predict U_Osm_, including Random Forest (RF)^31^, Gradient Boosting Machines (GBM)^32^, Automatic Machine Learning (AutoML)^33^ using h2o package^34^, and XGBoost^35^ using XGBoost package^36^ (Supplemental Methods; Supplemental Fig. 1). Cross-validation and hyperparameters tuning were used to optimize the ML models (Supplemental Methods). These methods were selected due to their capability of automatically handling missing values. For example, XGBoost supports missing values by default, with branch directions for missing values are learned during training. In h2o package, missing values are interpreted as containing information and in tree-based techniques split decisions for every node are found by treating missing values as a separate category.

The highest performing methodology was identified by applying the aforementioned ML techniques on the train set. Once the algorithm was obtained by the application of these techniques, predictions of U_Osm_ were generated on the test set. Once the predictions on the test set were calculated, Mean Absolute Error (MAE) was used to compare the predicted values of U_Osm_ to the actual ones recorded in the dataset. In particular, MAE is defined as $\frac{1}{N}\sum_{i=1}^{N}\left|{y}_{i}-{\widehat{y}}_{i}\right|$, where y_i_ and ${\widehat{y}}_{i}$ indicate the ith actual value and the related predicted value of the response variable respectively (i.e., ${\widehat{y}}_{i}$ estimates the conditional mean of U_Osm_ given subject-specific information). It was chosen as it is in the same scale of the outcome and does not assign large weight to infrequent large errors. The highest performing methodology, chosen on the lowest prediction error was selected (XGBoost). The reliability of the model was tested via repeating five times the whole train-test split and model estimation. Then, the performances on the test set were re-assessed to confirm matching results (Supplemental Table 3). The XGBoost is a black box model, where the importance of each feature on U_Osm_ is not disclosed. To elucidate the relationships among these variables, several simulations were performed using Partial Dependence Plots (PDP)^32,37^ and Accumulated Local Effects (ALE)^38^ plots (Supplemental Methods).

### Optimization

The ML approach allowed to establish a relationship between the U_Osm_ of each participant with their anthropometric characteristics, food and fluid intakes. Once this relationship was described, an optimization procedure was implemented to generate personalized advice on the daily amount of water needed to reach the target U_Osm_ for optimal hydration (i.e. a U_Osm_ < 500 mOsm/kg). For each individual, all anthropometric and food parameters remain unchanged. The only variable that was modified in the reverse engineering phase is the amount of plain water, which indirectly affects total fluid intake. The augmented Lagrangian was used as optimization algorithm^39^, because it also offers the opportunity to consider eventual constraints of interest which could be useful to avoid unreliable suggestions for particular individuals. This method integrates eventual non-linear constraints into the objective function (U_Osm_ < 500 mOsm/kg), so that a penalty is added for any violated constraint. The following constraints were used: the minimum and maximum acceptable values were set to 375 mOsm/kg and 625 mOsm/kg, respectively, and the target value was 500 mOsm/kg; the lower and upper bounds for the plain water intake were set to the observed minimum and maximum values in the dataset (i.e. 0 and 4050 mL); all other variables remained constant during the optimization process. This objective function was then optimized by a local solver without non-linear constraints using Sequential Quadratic Programming (SLSQP) algorithm^40^. The optimization procedure was applied on all the datapoints included in the test set.

### Evaluation of classification performance

Contingency tables were used to evaluate the classification rate of the developed algorithm (Table 1). The difference between the actual Total Fluid Intake (TFI) and the predicted TFI were calculated and checked against the actual U_Osm_ reported in the dataset, either above or below the 500 mOsm/kg threshold. This U_osm_ threshold is the target value of a 24 h urinary concentration and reflective of optimal fluid balance. This allowed to classify datapoints into four categories. True positives were those both with a higher TFI than the predicted TFI and being well hydrated (U_Osm_ < 500 mOsm/kg). True negatives were those both with a lower TFI than the predicted TFI and being underhydrated (U_Osm_ ≥ 500 mOsm/kg). The false positives were those with a higher TFI than the predicted TFI but who were underhydrated (U_Osm_ < 500 mOsm/kg). Which means that the predicted TFI is not high enough to ensure optimal hydration. Finally, the false negatives were those with a lower TFI than the predicted TFI but who were still considered well hydrated (U_Osm_ < 500 mOsm/kg). This means that the predicted TFI could have been lower.

**Table 1:** Evaluation of the algorithm classification.

	U_Osm_ < 500 mOsm/kg	U_Osm_ ≥ 500 mOsm/kg
∆[Actual-Predicted] Total fluid intake ≥ 0	True positive (TP)	False positive (FP)
∆[Actual-Predicted] Total fluid intake < 0	False negative (FN)	True negative (TN)

Overall percent classification by the algorithm was calculated using two metrics as follows:

$$Accuracy \, = \, \left( {TP \, + \, TN} \right) \, / \, \left( {TP \, + \, FP \, + \, FN \, + \, TN} \right),$$

$$Acceptable\,classifications \, = \, \left( {TP \, + \, FN \, + \, TN} \right) \, / \, \left( {TP \, + \, FP \, + \, FN \, + \, TN} \right).$$

The performance of the algorithm was further evaluated against values deriving from current European dietary guidelines for water intake. EFSA has set daily Adequate Intakes (AIs) for total water for the adult population of 2.5 L for men and 2.0 L for women^41^. These values include water that comes both from consumed fluids and food. It is estimated that the contribution of food and fluids to total water intake represent about 20% and 80%, respectively, in adults. This means male adults should drink 2.0 L per day, and female adults 1.6 L. These sex-specific thresholds were used to evaluate the difference between the actual TFI and the predicted TFI in relation to U_Osm_. The difference between the actual TFI and the EFSA AI was then checked against the actual U_Osm_ on the two sides of the 500 mOsm/kg threshold to generate contingency tables.

## Results

### Demographic characteristics

Data are presented as mean (min; max) or as % of the respective dataset, unless specified otherwise.

Both the train and test set showed an average age of 30 (19; 51) years (Supplemental Fig. 2) with a higher presence of female participants (64% and 62%, respectively). BMI was of 23 (18;30) kg/m^2^ in both sets. The range of U_Osm_ was [109–1252] mOsm/kg, with a mean value of 554 mOsm/kg in the train set, while in the test set it was [141–1343] mOsm/kg with a mean value of 546 mOsm/kg. The total fluid consumption showed an average value of about 1889 mL and a minimum value of 215 mL and 329 mL in the train and test sets, respectively. The maximum values were equal to 6109 mL and 6742 mL in the train set and test set, respectively. The total plain water consumption could vary between 0 and 4050 mL in the train set and between 100 and 3500 mL in the test set. The average values were about 1301 mL and 1049 mL, in the train and test sets, respectively.

### Feature selection

**Figure 2:**
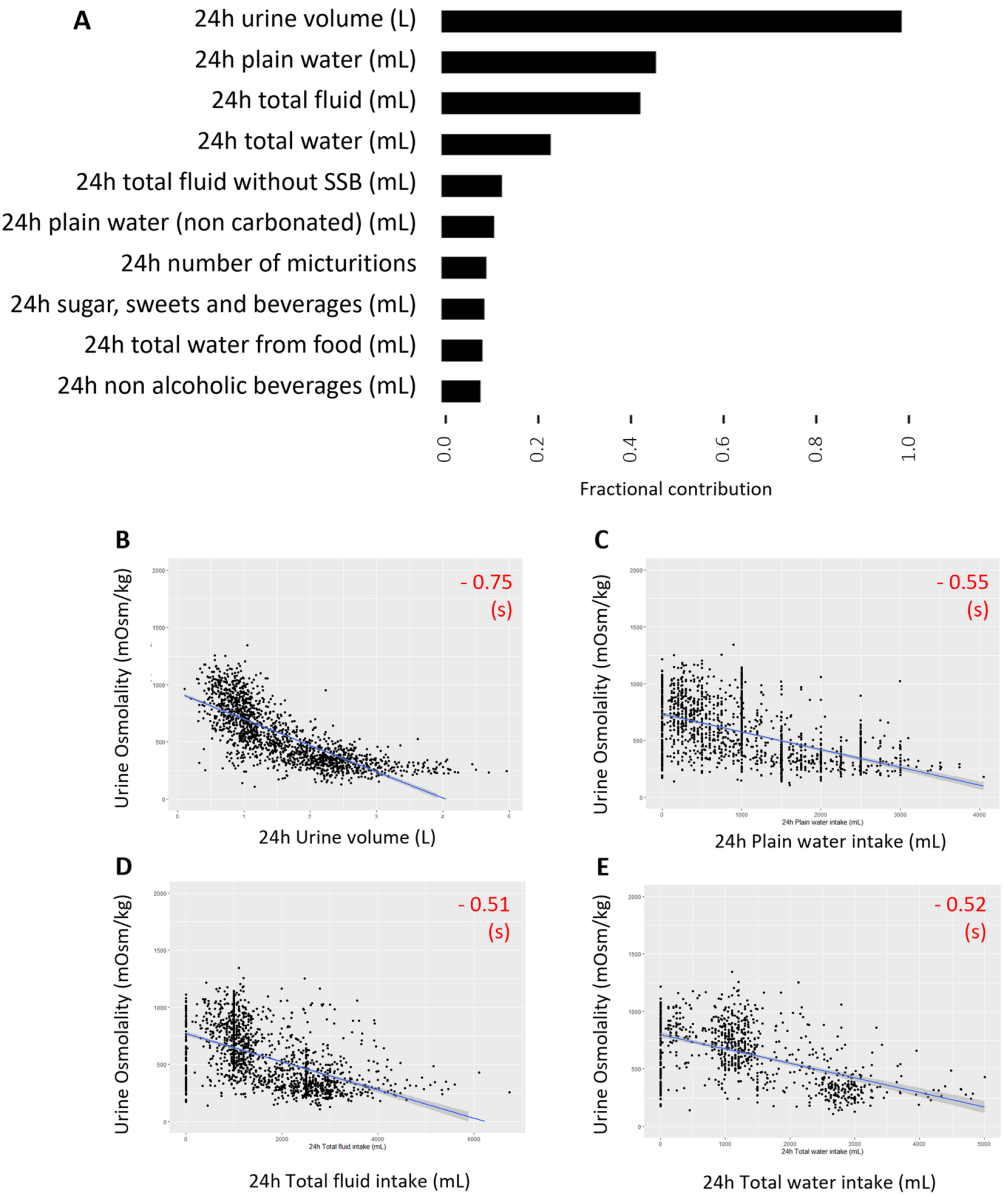
Variable importance to determine U_Osm_ and data distribution. (**A**) Feature importance plot showing the 10 most important features obtained with XGBoost. (**B**–**E**) Data distribution of Urine osmolality against 24 h Urine volume (n = 1575), Plain water intake (n = 1404), Total fluid intake (n = 1468) and Total water intake (n = 1448). Pearson’s correlation coefficient; (s) significant, (ns) non-significant based on linear regression p-value (< 0.05). The R ggplot2 package was used to generate the figures https://ggplot2.tidyverse.org. R Statistical Software version 3.6.3 (R Core Team https://cran.r-project.org/bin/windows/base/old/3.6.3/).

The preliminary identification of the most relevant features linked to urine production is shown in Fig. 2. The features are ranked based on their fractional contribution to the model, i.e. the total gain of each feature's splits (x-axis of Fig. 2A). The adopted configuration of the model is reported in Supplemental Methods. Features linked to urine production (volume and number of micturitions) as well as fluid consumption were listed among the most important features with relative importance values ranging from 0.10 to 0.95. At this stage, some key features were purposely excluded before generating predictions of urine osmolality such as Urine volume and Number of micturition, to only utilize information easily accessible to the general population. As a result, 23 variables out of 107 were kept in the dataset to generate predictions. These included Age, Sex, Weight, Height and some food and fluid intake variables (See Supplemental Table 2 for the full list of features).

### Mean Absolute Error (MAE) of machine learning algorithms

Several ML approaches were compared to generate predictions of urine osmolality. XGBoost was the most performing approach (MAE = 124.99), returning the minimum test error as compared to AutoML (MAE = 126.86), RF (MAE = 129.03) and GBM (MAE = 132.15). The results of the multiple evaluations of our models are reported in Supplemental Table 3. The average MAEs were 124.47, 126.88, 128.8, and 130.11 and the related standard deviations were 2.65, 1.38, 1.93, and 1.48, respectively.

**Figure 3:**
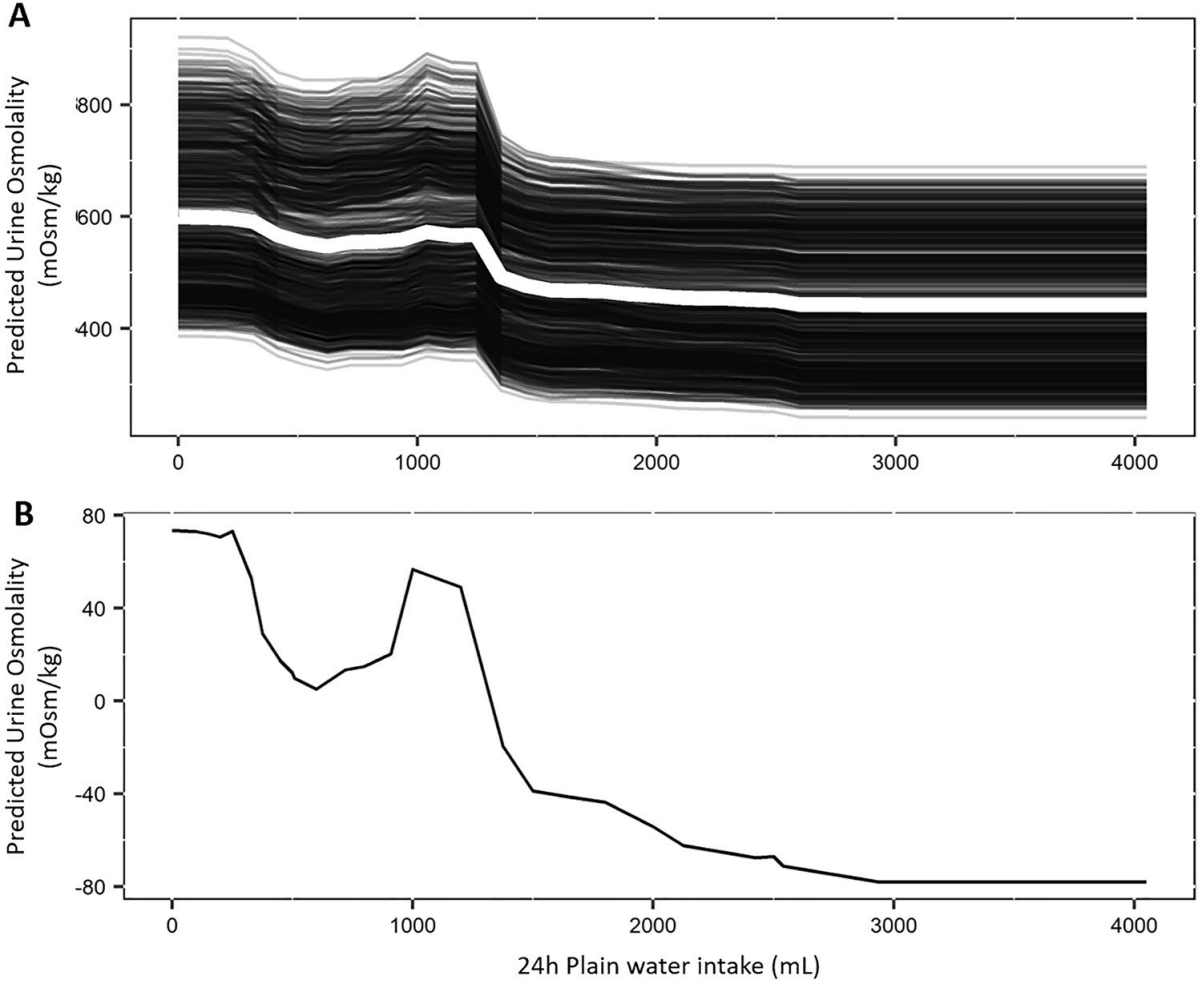
Partial dependence plots **(**PDP) and accumulated local effects (ALE) plots for 24 h Plain water intake. (**A**) PDP plot for 24 h plain water intake. The large white curve represents the PDP plot. The black lines represent the individual conditional expectation (ICE) curves, i.e. the equivalent to a PDP for individual data instances. (**B**) ALE plots for 24 h plain water intake. The R pdp package was used to generate the figures https://journal.r-project.org/archive/2017/RJ-2017-016/index.html. R Statistical Software version 3.6.3 (R Core Team https://cran.r-project.org/bin/windows/base/old/3.6.3/).

Since XGBoost is a black box model, several simulations were performed using PDP and ALE plots. Both methods showed that increasing water intake led to a progressive reduction of U_Osm_; the greater reduction was observed when moving from 1200 to 1500 mL (Fig. 3). The observed relationship between plain water intake and U_Osm_ reflected homeostatic fluid balance physiological processes.

An optimization algorithm was generated to frame the final advice for water intake, using constraints on 24 h U_Osm_ and 24 h plain water intake. The minimum and maximum acceptable values were set to 375 mOsm/kg and 625 mOsm/kg, respectively, and the target value was set to 500 mOsm/kg. The lower and upper bounds for the plain water intake were set to the observed minimum and maximum values from the dataset (i.e. 0 and 4050 mL). This allowed to estimate personalized advice for the individuals in the test set (Table 2). When the original urine osmolality was higher than 500 mOsm/kg, the tool calculated increased volumes of water intake. Conversely, when urine osmolality was low, (i.e. when the urine is diluted), the tool showed that the individual could reduce the water intake and still be optimally hydrated.

**Table 2:** Personalized advice for some individuals of the test set.

Age (years)	Height (cm)	Weight (kg)	Sex	Urine osmolality (mOsm/kg)		Plain water intake (mL)		Total fluid intake (mL)	
Original	Original	Original	Original	Original	Optimized	Original	Optimized	Original	Optimized
20	161	67	Female	1139	492	500	1298	1200	1998
42	160	67	Female	1020	505	250	1313	1000	2063
20	178	68.3	Male	750	501	1500	1750	2172	2422
26	181	75.2	Male	330	498	2000	1298	4115	3413

The algorithm developed in this study consisted of the combination of two main elements. Firstly, a ML algorithm, which generates a prediction for U_Osm_ starting from anthropometric characteristics, food, and fluid intake data. Secondly an optimization algorithm that generates personalized advice for optimal water intake based on the predicted U_Osm_ and actual fluid intake. To compare the performance of this combination of algorithms against current dietary guidelines, the relevance of the classification rates was calculated using contingency tables (Tables 3 and 4).

**Table 3:** Contingency table of the machine learning/optimization algorithm prediction of total fluid intake.

		U_Osm_		Accuracy	Acceptable classification
		< 500 mOsm/kg	≥ 500 mOsm/kg		
∆[Actual-predicted] Total fluid intake	≥ 0	185	22	85.5%	94.8%
	< 0	40	180		

Values are number of datapoints per category. Total sample size is *n* = 427 datapoints.

**Table 4:** Contingency table of values for total water intakes coming from fluids deriving from the European Food Safety Authority (EFSA) dietary guidelines.

		U_Osm_		Accuracy	Acceptable classification
		< 500 mOsm/kg	≥ 500 mOsm/kg		
∆[Actual-EFSA AI] Total fluid intake	≥ 0	161	31	77.8%	92.7%
	< 0	64	171		

Values are number of datapoints per category. Total sample size is n = 427 datapoints.

*EFSA AI* European Food Safety Authority Adequate Intake.

The developed algorithm exhibited the highest overall correct classification rates with an accuracy rate of 85.5% compared to an accuracy rate of 77.8% derived from dietary guidelines. When considering the proportion of false negatives, (i.e. participants with a lower fluid intake than the predicted one and showing a urine concentration below the threshold for optimal hydration) the rate of acceptable classifications was still higher for the algorithm developed in the current study (94.8%) than that derived from dietary guidelines (92.7%).

## Discussion

In this study, we generated a ML algorithm which in combination with an optimization algorithm provides personalized advice for daily water intake to achieve optimal hydration, as defined by a target 24 h U_Osm_ of 500 mOsm/kg in healthy adults. This target has been previously proposed to decrease risks for long term diseases while ensuring optimal fluid balance processes^19^. It is worth mentioning that we initially considered analyzing a subsample of data from the National Health and Nutrition Examination Survey (NHANES), a broad, validated and publicly available dataset of the United States population. Urine osmolality measures are available from NHANES 2009–2010 and 2011–2012. However, the large number of missing values and the low fractional contribution of the most relevant features to the model impaired the ability of generating predictions with an adequate degree of precision. All details about this analysis can be found in the Supplemental Data, NHANES section. Therefore, in the investigation here presented, we performed a post-hoc analysis of data pooled from multiple clinical studies on hydration and fluid balance in general adult population. This allowed to gather higher quality data from trustworthy and reliable sources in regards of data quality. Hence, we could highlight existing relationships between the observed features and U_Osm_. With no similar ML application reported in the literature, we evaluated the performance of the prediction against values deriving from current EFSA dietary guidelines for water intake. Our prediction model proved to generate advice for 24 h optimal water intake for healthy adults with an excellent degree of fitting compared to current adequate intakes (AIs) for water.

Representing 40–60% of body mass, water is the largest constituent of the human body. Like any other human physiological processes, water homeostasis is continuously challenged; in particular trans-epidermal, respiratory, fecal and urinary represent the main fluid losses and therefore threats to normal body functioning^6,42^. The human body has a limited capacity to store water and previous reports highlight that there is a mean daily water turnover of 3.6 ± 1.2 L/day or 2.8–3.3 and 3.4–3.8 L/day in women, and men, respectively^43^. Being between 0.25 to 0.35 L/day, only a fraction of water is produced in the body as result of metabolic processes, consequently, water losses must be replaced by ingestion of fluids^44^. The body puts in place highly refined responses to maintain the volume of the body water within a narrow range independently to the conditions that it may be facing^45^. Under this light, water has been called the ‘most essential’ nutrient^45^.

Despite its primary role, water is under-researched and often referred to as a forgotten, neglected nutrient. As an example, a review from Perrier and colleagues points to the discrepancies between regional water intake recommendations, and the fact that these reference values represent AIs^41,46,47^. The AIs derive from observational or experimental data that provide estimates of average water intake while lacking scientific evidence to associate a consumption threshold with positive or negative health outcomes^4,7^. From a practical point of view, general population currently does not benefit from recommendations for plain water intake in the dietary guidelines that are based on the health outcomes associated with the consumption of such nutrient. While thirst is generally considered enough of a warning for fluid replenishment^48,49^, guidelines are mostly generated for those who face physical exertion or extreme environmental conditions^50^. Implicitly, the message spread is that there is no need to pay attention to water intake except for those conditions.

On the contrary, a large and growing body of evidence suggests that maintaining an optimal water intake which avoids urine supersaturation and reduces excessive arginine vasopressin (AVP) secretion may be greatly beneficial for the kidney and reduce metabolic risk^47^. If we consider the astonishing increase in incidence of these diseases in the general population, this should be regarded as a form of primary prevention for public health. Ultimately, this optimal intake is reflected by the cut-off value of 500 mOsm/kg for 24 h U_Osm_^51–53^. Initially, this target was based on retrospective analyses of existing clinical data. Currently, evidence deriving from several randomized control trials have showed that reaching such a target for U_Osm_ can reduce circulating copeptin, as a proxy for AVP, as well as improve metabolic markers and reduce UTI incidence^3,16,22^.

In this investigation, we showed that a ML algorithm which integrates clinical features in combination with an optimization algorithm can accurately predict personalized water intake to achieve a target U_Osm_ of 500 mOsm/kg in healthy adults. More in detail, the algorithm holistically considers different variables spanning from anthropometric characteristics, biological, nutritional and beverage intake data. These variables may not be related to fluid-balance processes directly but comprehensively describe individuals from a physiological point of view. From there, it employs a data-driven unbiased approach to infer the main factors predictive of optimal water intake. To this instance, the algorithm identifies multiple functional pathways in respect to optimal water intake. As examples, individuals with low water intakes are associated with a higher U_Osm_ which results in advice to increase plain water consumption. Oppositely, individuals with low U_Osm_ are advised an amount of water which is lower compared to the reported data contained in the dataset. Therefore, when an individual shows high U_Osm_, the algorithm is essentially capable to advise an increase of plain water consumption, while when opposite scenario appears, a decrease in water consumption is proposed.

One of the first steps of our approach consisted in the ranking of the most important features related to urine osmolality. This revealed that the most important features were related to hydration physiology (i.e., urine volume, and the consumption of plain water and fluids in general). This shows that the ML approach is a suitable approach to model the fluids that come in and out of the human body. Urine volume and concentration are regulated by the same hormonal mechanisms and were shown to be highly correlated^3,54,55^. Given that the end goal of this study was to provide any human being with advice for water intake to be optimally hydrated, we had to consider the accessibility of the data that individuals could provide. The number of features was reduced to a minimum to allow for a minimalist need of information from the general population while maintaining and excellent output quality. As an example, outside of any clinical context, people would generally be able to provide anthropometric variables such as age, sex, weight, and height. Also, food and fluid intake data can be easily recollected. Oppositely, the volume of urine collected throughout a 24 h period may pose some challenges and therefore prevent usage from the general population. For this reason, the algorithms developed do not ask for such information to generate a prediction for optimal water intake.

Our investigation represents a first exploration on the application of ML techniques on fluid balance physiological processes. Nonetheless, the study here presented comes with several limitations. Currently, the overall weight in determining the prediction for water intake between the algorithm and the optimization process is undetermined. Future research should address this aspect to determine whether more effective methodologies in regards of the optimization algorithm could be implemented. However, it appears that with current optimization process the overall performance of the algorithm in providing a prediction for optimal intake remains adequate. For this initial investigation, the data used to generate predictions rely uniquely on a subset of healthy clinical trials’ participants. Athletes, or subject taking part in strenuous physical activity, pregnant and lactating women were excluded from these trials on purpose. Therefore, it should be regarded as a priority to integrate data from more diverse and vulnerable populations such as aging population^56,57^, pregnant and lactating women^58,59^, and people regularly drinking extremely low or high volumes of water, especially when their U_Osm_ still shows reasonable values regardless of the expectations. The latter would allow to test the method at the two-ends of the physiological spectrum. Integrating additional extrinsic data on seasonality, ambient temperature, physical activity should be regarded as an additional next step to further personalize the advice on water intake. Additionally, while considering different beverages consumed by individuals, the current algorithm only modulates plain water intake in the prediction generated. While water consumption is associated with positive health outcomes, the same cannot be currently supported for other beverages. Therefore, future developments could integrate advice for different beverages in combination with plain water intake. Finally, a clinical validation of the proposed ML algorithm is warranted to validate the predicted amount water to achieve U_Osm_ of 500 mOsm/kg with real world evidence.

An additional learning we would like to share is about the importance of blending high-quality, context-specific data deriving from clinical studies into ML. This may allow to fix the reliability issues often reported by healthcare recipients when using artificial intelligence derived applications in decision-making processes.

## Conclusions

Employing personalized advice for optimal water intake may contribute to decrease disease development and progression and may also be valuable in rationally designing nutritional interventions in a variety of kidney and metabolic disorders. More broadly, accurate personalized water intake predictions in these scenarios may be of great practical value, as they will integrate nutritional modifications more extensively into the clinical decision-making scheme. Indeed, we contributed to the demonstration that artificial intelligence and ML adoption can be implemented for taking advantage of digital algorithmic evidence to improve healthcare in general population. Here, we present an application of ML as primary prevention providing personalized advice on daily water intake considering personal intrinsic and extrinsic variability to achieve an optimal target for U_Osm_ of 500 mOsm/kg.

## Supplementary Information

Supplementary Information.

## Data Availability

The source code and datasets generated and analyzed during the current study may be made available from the corresponding author with prior agreement of legal and compliance Danone Research offices on reasonable request.

## Abbreviations

AIs: Adequate intakes
ALE: Accumulated local effects
AUC: Area under the curve
AutoML: Automatic machine learning
AVP: Arginine vasopressin
BMI: Body mass index
EFSA: European Food Safety Authority
FDA: Federal Drug Administration
GBM: Gradient boosting machines
IOM: Institute of Medicine
MAE: Mean absolute error
MEC: Mobile Examination Center
ML: Machine learning
NHANES: National Health and Nutrition Examination Survey
PDP: Partial dependence plots
P_Osm_: Plasma osmolality
RF: Random forest
SLSQP: Sequential quadratic programming
TFI: Total fluid intake
U_Osm_: Urine osmolality
US: United States
U_SG_: Urine specific gravity
UTI: Urinary tract infections
WHO: World Health Organisation
XGBoost: Extreme gradient boosting
