# Supplementary material for: Personalized prediction of optimal water intake in adult population by blended use of machine learning and clinical data
Source: Sci Rep. 2022 Nov 16;12:19692. doi: 10.1038/s41598-022-21869-y (PMC9669042; doi:10.1038/s41598-022-21869-y)
Supplement: Supplementary file 1 — Supplementary Information. [file 41598_2022_21869_MOESM1_ESM.docx]

Table of contents

[1. Supplemental Tables 2](#_Toc115167263)

[Supplemental Table 1. 2](#_Toc115167264)

[Supplemental Table 2. 3](#_Toc115167265)

[Supplemental Table 3. 4](#_Toc115167266)

[2. Supplemental Figures 5](#_Toc115167267)

[Supplemental Figure 1. 5](#_Toc115167268)

[Supplemental Figure 2. 6](#_Toc115167269)

[3. Supplemental Methods 7](#_Toc115167270)

[4. Supplemental data – NHANES 17](#_Toc115167271)

[Methods 17](#_Toc115167272)

[Results 20](#_Toc115167273)

[5. Reference List 23](#_Toc115167274)

# Supplemental Tables

Supplemental Table 1. % missing data for the main features

|  | **A** Variable | Missing values (%) CLN |
| --- | --- | --- |
|  | Age | 0.07 |
|  | Sex | 0 |
|  | Height | 0.07 |
|  | Weight | 0.07 |
|  | BMI | 0.07 |
|  | U_Osm_ | 0 |
|  |  |  |
|  | **B** Variable | Missing values (%) |
|  | 24h urine volume | 0 |
|  | 24h plain water | 0 |
|  | 24h total fluid | 0 |
|  | 24h total water | 48 |
|  | 24h total fluid without sugar-sweetened-beverages | 34 |
|  | 24h plain water (non carbonated) | 0 |
|  | 24h number of micturitions | 67 |
|  | 24h sugar, sweets and beverages | 34 |
|  | 24h total water from food | 59 |
|  | 24h non alcoholic beverages | 46 |

Supplemental Table 1: % of missing data for main features. Table **A** presents % of missing values for participants demographic characteristics. Table **B** presents % of missing values for for top 10 features to determine U_Osm_ after filtering.

Supplemental Table 2. Full list of features considered

| Variable | Description | Unit |
| --- | --- | --- |
| SEX_COD | Sex | / |
| AGE_NUM | Age | year |
| HEIGHT_V1 | Height | cm |
| WEIGHT_V1 | Weight measured during the "V1" clinical visit | kg |
| VS_WEIGHT | Weight measured during the "VS" clinical visit | kg |
| ML_FOOD_COD_LEV1_1 | Milk and milk products | mL |
| ML_FOOD_COD_LEV1_2 | Meat, poultry, fish, and mixtures | mL |
| ML_FOOD_COD_LEV1_3 | Eggs | mL |
| ML_FOOD_COD_LEV1_4 | Dry beans, peas, other legumes, nuts, and seeds | mL |
| ML_FOOD_COD_LEV1_5 | Grain products | mL |
| ML_FOOD_COD_LEV1_6 | Fruits | mL |
| ML_FOOD_COD_LEV1_7 | Vegetables | mL |
| ML_FOOD_COD_LEV1_8 | Fats, oils, and salad dressings | mL |
| ML_FOOD_COD_LEV1_9 | Sugars, sweets, and beverages | mL |
| cm_24H_TOTAL_FLUID_CONSUMPTION | 24h total fluid | mL |
| cm_24H_PLAIN_WATER_CONSUMPTION | 24h plain water | mL |
| cm_24H_DAIRY_BEVERAGE_CONSUMPTION | 24h dairy beverages | mL |
| cm_24H_SSB_CONSUMPTION | 24h sugar sweetened beverages | mL |
| cm_24H_NON_SSB_CONSUMPTION | 24h total fluid without SSB | mL |
| cm_24H_HOT_BEVERAGES_CONSUMPTION | 24h hot beverages | mL |
| cm_24H_ALCOHOL CONSUMPTION | 24h alcoholic bevergaes | mL |
| cm_24H_TOTAL_WATER_FROM_FOOD_CONSUMPTION | 24h total water from food | mL |
| cm_24H_TOTAL_WATER_CONSUMPTION | 24h total water | mL |

SSB: Sugar-sweetened-beverages

Supplemental Table 3. Multiple models evaluation – MAE (Mean Absolute Error)

|  | XGBoost | AutoML | RF | GBM |
| --- | --- | --- | --- | --- |
| Run1 | 124,99 | 126,86 | 129,03 | 132,15 |
| Run2 | 126,45 | 125,92 | 130,01 | 130,49 |
| Run3 | 119,92 | 128,67 | 130,78 | 130,53 |
| Run4 | 124,73 | 125,21 | 125,76 | 128,32 |
| Run5 | 126,24 | 127,74 | 128,42 | 129,07 |

# Supplemental Figures

Supplemental Figure 1. Flowchart ML model estimation and optimization.


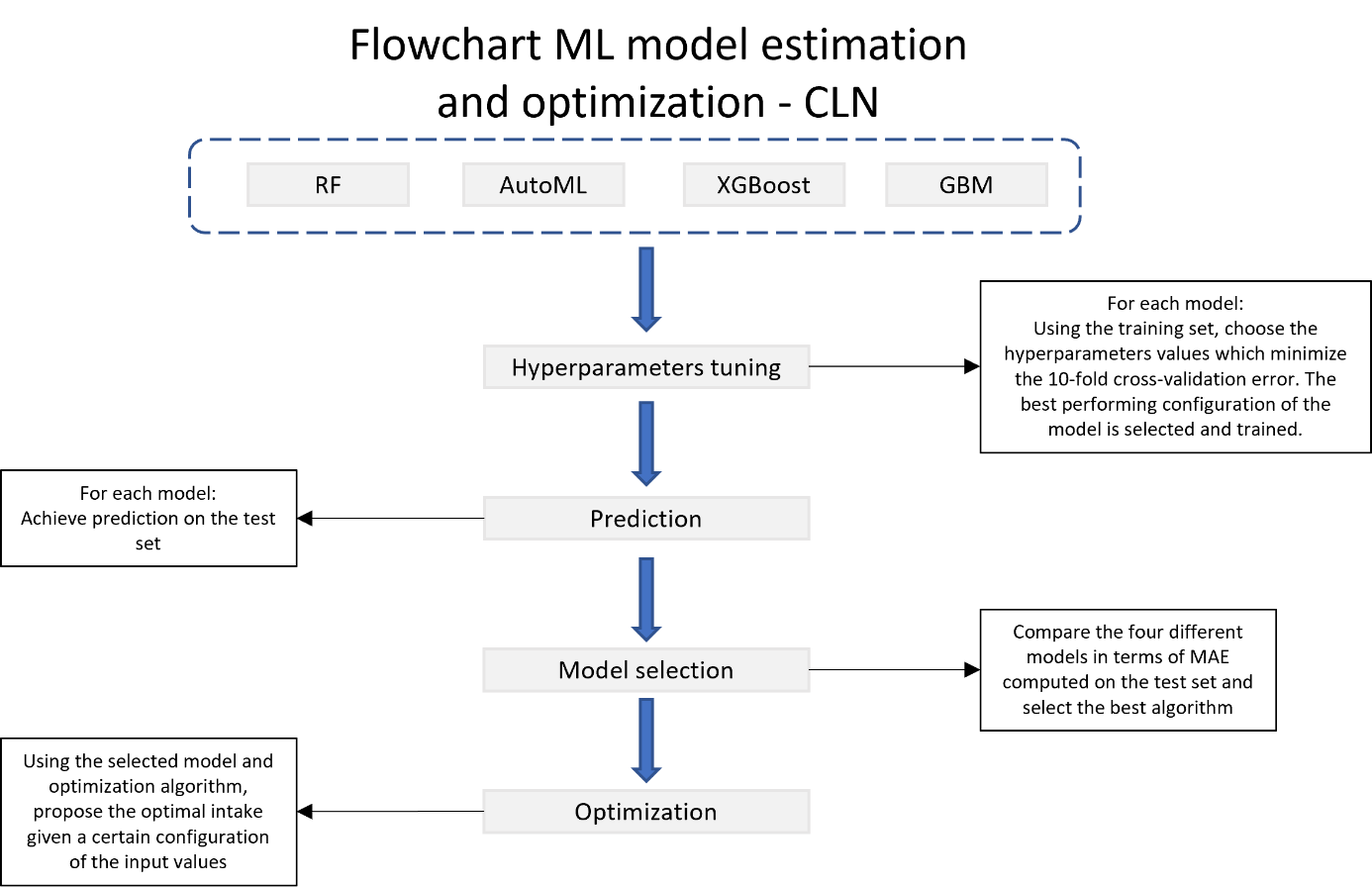


Supplemental Figure 2. Comparison of train and test set.


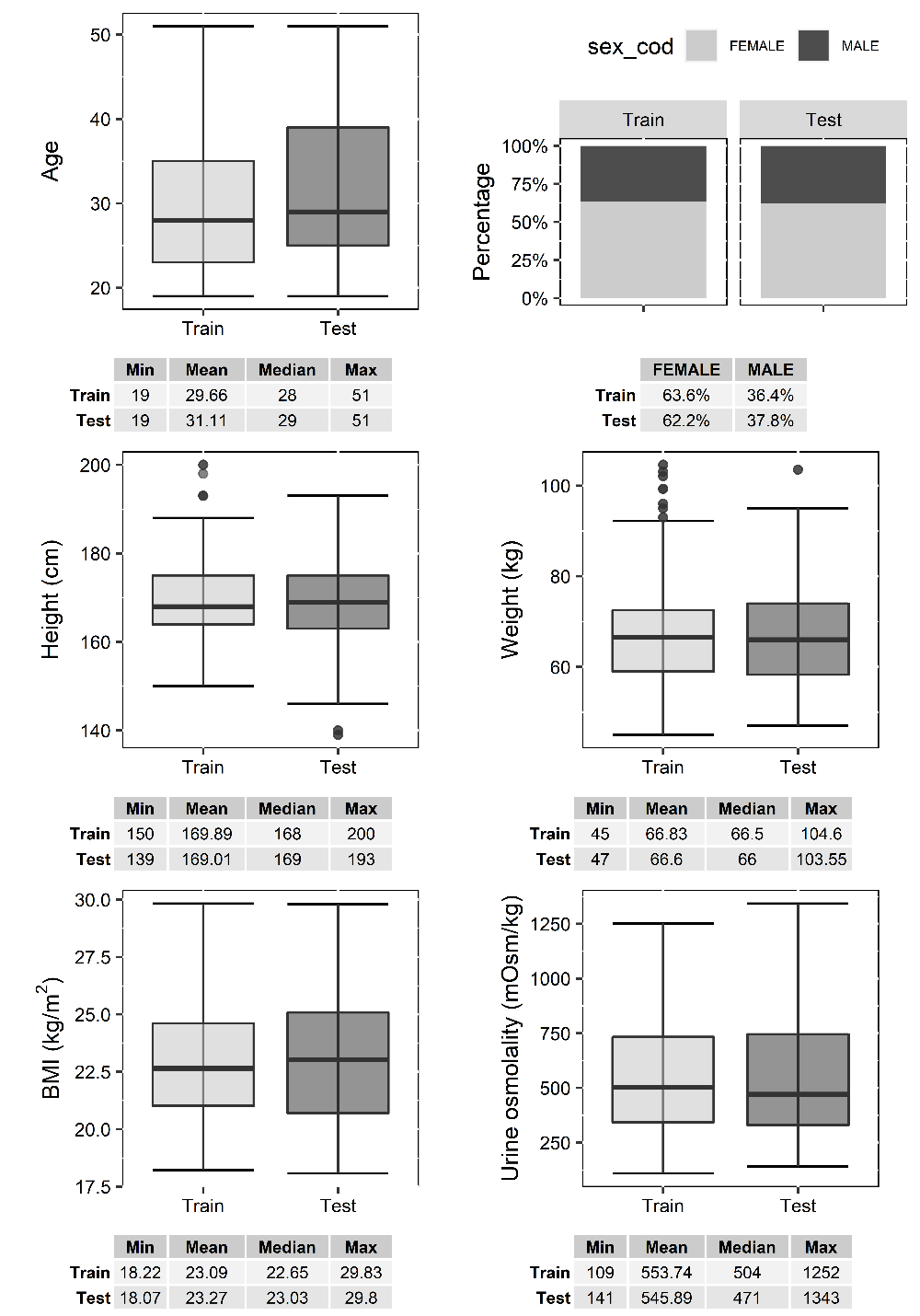


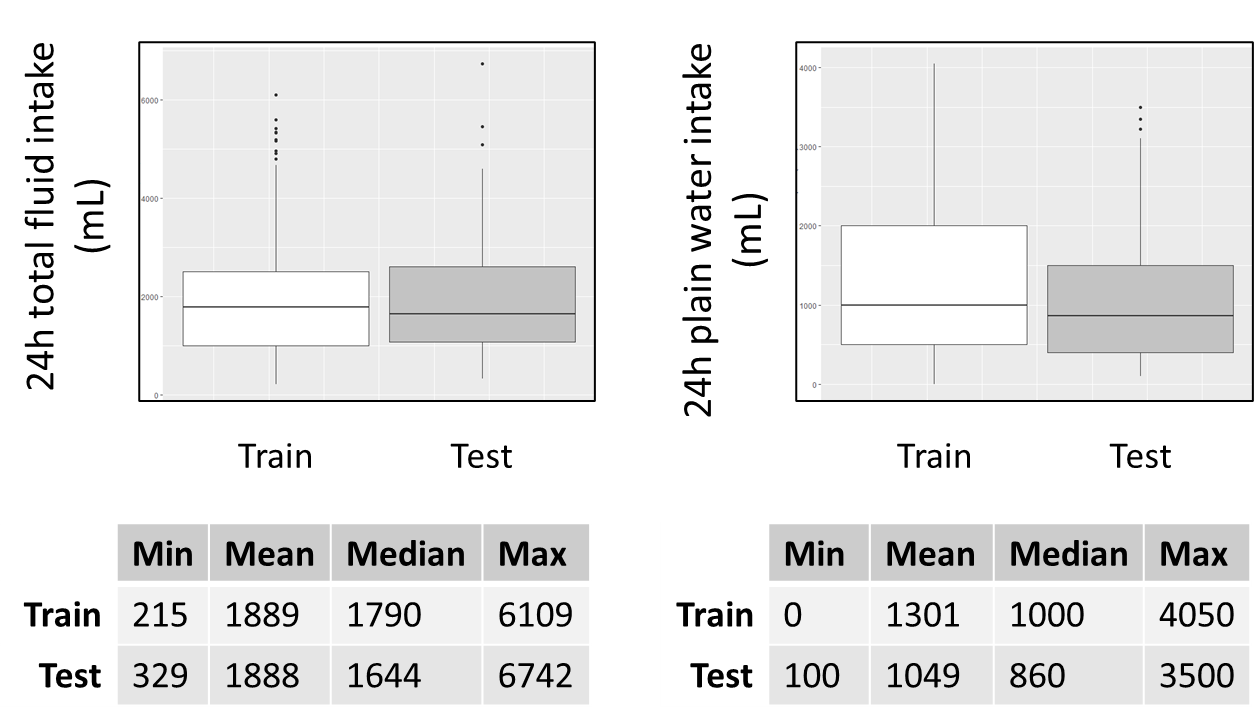


# Supplemental Methods

**Clinical trials’ information**

| **Study name, year, objective** | **Ethics Committee** | **Identification number** | **Extract of Inclusion criteria (anthropometry, hydration physiology, health, exercise)** | **Extract of exclusion criteria (anthropometry, hydration physiology, health, exercise)** |
| --- | --- | --- | --- | --- |
| Effect of increasing water intake by 2 litres per day on the crystallization risk index in healthy subjects (2008) | - Comité Etico De Investigacion Clinica (CEIC) del Hospital Universitario La Princesa, Madrid, Spain  - CEIC Hospital Universitario de La Paz, Madrid, Spain | N° EudraCT: NA | - Healthy male and female subjects, aged 25 to 50 years (limits included), with a urine osmolality > 450 mosmol/Kg for women and > 550 mosmol/Kg for men and with an osmotic load > 10 mosmol/kg BW/d based on a 24H urine collection confirmed by creatinine clearance > 90mL/min, with a body mass index between 20 and 30 kg/m2 (bound 20 included, bound 30 excluded)  -Subject not taking any treatment for anorexia, weight loss, or any form of treatment likely to interfere with metabolism or dietary habits  -Female subject using an efficient contraceptive method | - Subject having experienced one or more lithiasis event; with a history of metabolic or gastrointestinal disease with the exception of appendicectomy; undergoing general anaesthesia in the month prior to inclusion; receiving systemic treatment or topical treatment likely to interfere with evaluation of the study parameters; presenting chronic infection requiring antiseptic/antibiotic treatment more than three times in one year; with renal failure; with hepatic failure; with cardiac failure; with chronic or iatrogenic immunodepression; taking antibiotics during the last months  - For female subjects: pregnancy or breast feeding  - Subject should not engage in intense physical activity (e.g. marathon training & body building) |
| Effect of increasing water intake by 2 litres per day on the crystallization risk index in healthy subjects (2008) | - Comité de Ética e Investigación para Estudios en Humanos (CEIEH), Mexico, Mexico  - Comité de Bioética Para la Investigacion Clinica, Mexico, Mexico | N° EudraCT: NA |  |  |
| Evaluation of hydration markers  in French adult population | Comité de Protection des Personnes (CPP) of Ile de France XI, Paris, France | N° ID RCB: 2009-A00541-56 | - Generally healthy male and female subject, aged from 25 to 40 years old (bounds included), with a body mass index 18.5 and 29.5 kg/m^2^ (bounds included);  - Subject with negative results at the serology test concerning HIV, Hepatitis B & C  - For female subject: using an efficient contraceptive method  - Caucasian subject with globally French dietary habits to minimize strong interindividual variations in kidney morphology and physiology that can occur between populations adapted to different environment, particularly to 2 environments  - Subject accepting not to perform exercise during the study period  - Subject considered as low drinker: with a total fluid intake below 1.2 Litter per day per 1.73m² of body surface area (BSA) or high drinker: with a total fluid intake between 2 and 4.0 litter per day per 1.73m² of BSA (bounds included) | - Subject under medication likely to interfere with metabolism (in particular hypotensive treatment, diuretic for renal function, …); with a history of metabolic disease or with acute or chronic gastrointestinal or metabolic disease (such as diabetes mellitus); undergoing general anaesthesia in the month prior to inclusion; receiving systemic treatment or topical treatment likely to interfere with evaluation of the study parameters; presenting chronic infection requiring antiseptic/antibiotic treatment more than three times in one year or taking antibiotics currently or during the last month; with renal, hepatic or cardiac failure  - Subject practising physical activity in an intensive way  - Subject consuming more than 15 cigarettes per day; consuming more than 2 units of alcohol for women and more than 3 units of alcohol per weekday  - For female subject: pregnancy; breast feeding |
| Effect of an increased water intake on the DNA adducts formation associated with tobacco in smokers (2011) | Ethics Committee CPP Sud Méditerranée III, Nîmes, France | N° ID RCB: 2010-A01488-31  ClinicalTrials.gov Identifier: NCT01583387 | - Male subjects 20 to 45 years old (bound included), with body mass index between 18 and 27kg/m2 (bound included)  - Smokers with a moderate or high level of dependence to cigarette according to the score Fagerstrom test (score ≥ 5), with at least 15 cigarettes/day for the last 2 years  - Small drinkers (fluid intake ≤1L of fluid and ≤500 ml of water per day) as assessed during the pre-screening  interview | - Subjects presenting evidence or history of severe or acute disease which could affect the results of the study or the vital status; with a history of metabolic disease or with acute or chronic gastrointestinal disease except appendicectomy; with diagnosed urinary tract disease; with diagnosed lung or respiratory disease; under local or general treatment which can modify measurements performed in the study, in particular the assessment of the hydration status (diuretic intake, or treatment interfering with metabolism  and nutrition behaviour); presenting a situation interfering with the outcomes of the study according to the investigator  opinion  - Subject practising physical activity in an intensive way according to the investigator judgment.  - Subjects consuming regularly more than 3 units of alcohol per day; taking drugs or presenting drug addiction (cannabis, opioids, amphetamines.)  - Subjects not able or not wishing to increase their fluid intake by 1.5L per day during the evaluation phase  - Subjects planning to stop or reducing smoking or change their cigarette brand within the next 3 months |
| Evaluation of circadian variations of saliva osmolality in healthy young subjects.  (2011) | Ethics Committee CPP Est IV, Strasbourg, France | N° ID RCB: 2010-A01005-34 | - Female and male subjects aged 20-30 years old (both ages included), with a Body Mass Index (BMI) within the range 20-25 kg/m^2^ (both inclusive), with a height within the range 1.60-1.75 m (both inclusive) for female subjects and 1.70-1.85 m (both inclusive) for male subjects.  - Subjects must be in good health as determined by their medical history, physical examination, ECG, vital signs and laboratory tests  - For female: Subjects with monophasic contraceptive method.  - Subjects considered as **low drinker**: females with a total fluid intake below 1.2L per day; males with a total daily fluid intake below 1.2L per day; **high drinker**: females with a total fluid intake between 2 and 4L per day (bounds included)  - Subjects must refrain from intensive physical exercise during the study conduct (from the screening visit until the end of study visit).  - Subjects who do not smoke. | - Use of any prescription or over-the-counter medication within 14 days prior to admission (not including paracetamol in the range of 1.5 gr/day and monophasic contraceptive therapy for female); any medications with central effects are prohibited for a period equal to 5 x half-life prior to admission (D0), should this period be longer than 14 days; any clinically relevant acute or chronic diseases which could interfere with the subjects safety during the trial, or expose them to undue risk, or which could interfere with the study objectives; likely to need any treatment (including dental care) during the study period; positive test result on hepatitis B surface antigen, hepatitis C antibody; positive test result on HIV 1 and 2 serology.  - Excessive daily consumption of xanthines containing drinks (i.e > 250 mg/day of caffeine).  - Pregnant females as determined by a pregnancy test or breast feeding females.  - Alcohol consumption > 20 g alcohol/day;  - History of substance abuse, known drug addiction, or positive test for drugs of abuse. |
| Validation of spot urine as a biomarker of fluid intake in real life conditions.  (2013) | Ethics Committee CPP Est III, Nancy, France | N°ID RCB: 2012-A01694-39  ClinicalTrials.gov Identifier: NCT02044679 | - Free living female and male subjects aged 20- 30 years old (both ages included), with a BMI within the range 20-25 kg/m2 (both inclusive), with a height within the range 1.60-1.75 m (both inclusive) for female subjects and 1.70-1.85 m (both inclusive) for male subjects.  - Subjects in good health as determined by medical history, physical examination, vital signs (body weight, blood pressure, heart rate) and laboratory tests for exclusion criteria  - For female: Subjects with monophasic contraceptive method.  - Subjects who agree to refrain from intensive physical exercise during the study period (from the screening  visit until the end of study visit).  - Subjects smoking less than 10 cigarettes/day. | - Use of any prescription or over-the-counter medication within 14 days prior to admission (not including paracetamol in the range of 1.5 g/day and monophasic contraceptive therapy for females); any medications with central effects are to be prohibited for a period equal to 5 x half-life prior to admission, should this period be longer than 14 days; any clinically relevant acute or chronic diseases which could interfere with the subjects’ safety during the trial, or expose them to undue risk, or which could interfere with the study objectives; having participated in a clinical study for the renal diseases or having received any treatment related to the kidneys, cardiovascular disease or to hypertension in the last 12 months; likely to need any treatment (including dental care) during the study period.  - Pregnant woman or woman planning to become pregnant during the study; breast-feeding woman.  - Alcohol consumption > 20 g alcohol/day  - History of substance abuse, known drug addiction, or positive test for drugs of abuse. |
| The effect of increased water intake on the frequency of clinical recurrent urinary tract infections in pre-menopausal women  (2016) | Ethics Committee of COMAC Medical, Sofia, Bulgaria | ClinicalTrials.gov Identifier: NCT02444975 | - Women with clinical recurrences of symptomatic urinary tract infections (UTI), aged ≥ 18 years  -Women using any form of contraception  - Fluid intake < 1.5 L per day  - Women accepting to keep their lifestyle habits during the whole duration of the study | - Women with history of UTI complications (pyelonephritis or other) in the last 12 months; use of antibiotics or cranberries juice and / or extracts in the previous 2 weeks; chronic treatments with anti-coagulants therapy; chronic bladder inflammation; chronic diarrhea or constipation treated with chronic use of laxative substances; interstitial cystitis; estrogen-dependent symptomatic vulvo-vaginitis; recent ( <1year) or active renal stone disease; urinary tract structural abnormalities; on-going or planned therapy during the study which can modify the study measurements, in particular the assessment of the hydration status (diuretic intake, corticoids or drug treatment interfering with nutrition behaviour); subjects with severe or uncontrolled organic disease, likely to interfere with the parameters of the study (e.g. neoplastic, cardiovascular, pulmonary and digestive disorders, unstabilised diabetes type I and II, untreated or uncontrolled clinically significant arterial blood hypertension) or mental disorders affecting eating and drinking behaviour (i.e. primary polydipsia, bulimia nervosa, psychosis, etc.); Women who have taken part in any other clinical study for the treatment of rUTI during the last 12 months  - Obesity or malnutrition (BMI <18.5 Kg/m2 and >30 Kg/m2)  - Pregnant or lactating women; women planning to become pregnant during the study; menopausal and peri-menopausal women  - Incapacity / non-willingness to consume 1.5 L of drinking water per day on top of their usual consumption |

**Feature importance**

To order the available features according to their importance we decided to rely on a XGBoost model. Such an algorithm can indeed be adopted to compute the fractional contribution of each feature to the model based on the total gain of this feature's splits. More details about this specific ML algorithm are provided in the following section.

XGBoost hyperparameter tuning was applied on the dataset, using a grid search and a 10-fold cross-validation. The final hyperparameter configurations were:

| **Hyperparameter** | **Dataset** |
| --- | --- |
| ntrees | 765 |
| sample_rate | 0.7 |
| max_depth | 4 |
| min_split_improvement | 1e-6 |
| nbins | 16 |
| nbins_cats | 16 |
| min_rows | 16 |
| col_sampe_rate | 0.8 |
| col_sampe_rate_per_tree | 0.7 |
| learn_rate_annealing | 0.99 |
| learn_rate | 0.1 |

**ML algorithms**

Random Forest (RF) is a widely used tree-based machine learning technique. The rationale behind this method is that multiple regression trees are applied each on a different random subset of features and data points and their predictions are finally combined. This is a so-called ensemble method, given that it combines indications provided by simpler individual regression techniques.

Gradient Boosting Machine (GBM) is another popular machine learning that identify several rough prediction rules (i.e. weak learners) and combining them it is possible to improve predictive performances. Differently from RF, the regression trees are fitted sequentially, focusing, at each iteration, on the data points predicted poorly up to that moment. Extreme Gradient Boosting is essentially a modification of the standard GBM. The main differences are represented by the use of an advanced regularization to improve model generalization and the use of second-order gradients during the optimization of the loss function.

Finally, Automatic Machine Learning (AutoML) is a particular tool which automatically train multiple machine learning methods, namely Random Forest, Generalized Linear Model with regularization, Gradient Boosting Machine, Artificial Neural Network and ensembles of all the base models or using subsets of the base models.

All these methods allow to model complex relationships between explanatory variables and the outcome of interest, avoiding manual setting of eventual interactions between predictors; They provide immediate importance measures for the considered features. For these reasons, they were chosen for the current analysis.

For each ML methodology applied, hyperparameter tuning was applied using a grid search and a 10-fold cross-validation.

To apply RF on these data, we decided to tune several different hyperparameters and among the most relevant ones are:

- the number of trees in the forest (ntrees)
- the number of variables randomly selected for each tree (mtries)
- the row sampling rate (sample_rate)
- the maximum depth of each tree (max_depth)
- the minimum relative improvement in the error to allow a split to happen (min_split_improvement).

Additional considered hyperparameters provided by the h2o package were: nbins, nbins_cats, min_rows and col_sample_rate_per_tree.

Table below shows the optimal configuration achieved in the present analysis. The resulting RF provided a Mean Absolute Error (MAE) equal to 129.03.

**RF tuned hyperparameters**

| **Hyperparameter** | **Value** |
| --- | --- |
| ntrees | 100 |
| mtries | 5 |
| sample_rate | 0.5 |
| max_depth | 15 |
| min_split_improvement | 0 |
| nbins | 512 |
| nbins_cats | 1024 |
| min_rows | 4 |
| col_sampe_rate_per_tree | 0.75 |

Hyperparameters tuning was performed also for the use of GBM. In this case the search was focused on:

- the maximum depth of each tree (max_depth).
- the row sampling rate (sample_rate).
- the column sampling rate per split (col_sample_rate) and per tree (col_sample_rate_per_tree).
- the minimum relative improvement in the error to allow a split to happen (min_split_improvement).

Additional considered hyperparameters were: nbins, nbins_cats, min_rows, learn_rate and learn_rate_annealing. The maximum number of trees was set to 10000.

The optimal configuration is reported in table below. This GBM showed a MAE equal to 132.15.

**GBM tuned hyperparameters**

| **Hyperparameter** | **Value** |
| --- | --- |
| ntrees | 1147 |
| sample_rate | 0.6 |
| max_depth | 4 |
| min_split_improvement | 1e-6 |
| nbins | 16 |
| nbins_cats | 16 |
| min_rows | 16 |
| col_sampe_rate | 0.8 |
| col_sampe_rate_per_tree | 0.6 |
| learn_rate_annealing | 0.99 |
| learn_rate | 0.1 |

A grid search was then performed to identify the best configuration of the hyperparameters of the XGBoost model. We looked for the optimal values of:

- the learning rate (eta).
- the minimum loss improvement to perform a split (gamma).
- the maximum depth of each tree (max_depth).
- the row sampling rate (subsample).
- the minimum sum of instance weight in a child (min_child_weight).
- the column sampling rate per split (colsample_bylevel) and per tree (colsample_bytree).

The maximum number of rounds (nrounds) was fixed to 1000 and the early_stopping_rounds hyperparameter was fixed to 5 to prevent overfitting, stopping the training when the cross-validation error does not improve for 5 rounds. The hyperparameters tuning lead to the configuration reported in Table. The related XGBoost model produced a MAE equal to 124.99.

**XGBoost tuned hyperparameters**

| **Hyperparameter** | **Value** |
| --- | --- |
| ntrees | 1147 |
| sample_rate | 0.6 |
| max_depth | 4 |
| min_split_improvement | 1e-6 |
| nbins | 16 |
| nbins_cats | 16 |
| min_rows | 16 |
| col_sampe_rate | 0.8 |
| col_sampe_rate_per_tree | 0.6 |
| learn_rate_annealing | 0.99 |
| learn_rate | 0.1 |

Finally, AutoML method provided by *h2o* was also applied. This method performs automatic training and tuning of several ML models. It allows the users to specify the maximum number of models to be considered (max_models) and the maximum time to be taken to perform the search of the best ML model (max_runtime_secs). Max_models was set to 200 and max_runtime_secs to 3000.

The best model identified by this method was a Gradient Boosting Machine with the hyperparameters set to the values reported in Table. This model provided a Mean Absolute Error equal to 126.86.

**AutoML best model tuned parameters**

| **Hyperparameter** | **Value** |
| --- | --- |
| ntrees | 555 |
| sample_rate | 0.5 |
| max_depth | 8 |
| min_split_improvement | 1e-5 |
| nbins | 20 |
| nbins_cats | 1024 |
| min_rows | 10 |
| col_sampe_rate | 0.4 |
| col_sampe_rate_per_tree | 0.7 |
| learn_rate_annealing | 1 |
| learn_rate | 0.008 |

**PDP and ALE**

PDP is a model agnostic tool introduced by Friedman [1], which shows how the average prediction changes when the variable of interest X varies across a specified range. Given a certain grid of possible values of X, for each value x it shows the average value predicted by the ML model when setting X=x for each data instance. The main issue with PDP is the assumption of independence of the feature of interest with respect to the remaining ones. When this assumption is not satisfied the risk is to average predictions that are unlikely, leading to a great bias in the estimated feature effect.

To solve this problem, ALE plots [2] are commonly adopted. These plots average the differences in predictions for specific intervals into which the domain of X is divided. For each instance in a certain interval, the value of X is replaced by the upper and lower bounds of the interval and the difference in predictions is retained. The average difference is then calculated. The process is repeated for all the intervals and the average effects are accumulated, so that if x lies in the last interval, the ALE is the sum of the effects of all the intervals. The effects are then centered to have a mean effect equal to 0. A value of the ALE can be also seen as the effect of X = x compared to the average prediction of the data. Given a certain interval, using only instances having an original value similar to x allows us to avoid including unlikely instances.

# Supplemental data – NHANES

## Methods

**Study Population**

We first used de-identified data from the National Health and Nutrition Examination Survey (NHANES), a series of ongoing cross-sectional, population-based surveys implemented in the United States by the Centers for Disease Control and Prevention (CDC). NHANES is dedicated to monitor the health and nutritional status of the population using a stratified, nationally representative sampling of the non-institutionalized US civilian population. Detailed survey descriptions, methodology, sampling procedures, laboratory test procedures, and datasets are publicly accessible (www.cdc.gov.nchs/nhanes/). All NHANES participants have provided written consent for their information to be used in research. All protocols have received approval by the National Center for Health Statistics Research Ethics Review Board. NHANES waves from 1999 until present were screened for urine, blood, fluid, and food parameters. Waves 2009-2010 (*n* = 10,537) and 2011-2012 (*n* = 9,756) were the only two waves comprising assessments of U_Osm_ and were pooled together to obtain reliable estimates for optimal water intake. Physiological (self- reported pregnancy, *n* = 738 [NHANES 2009-2010]/ 570 [NHANES 2011-2012]; breastfeeding, *n* = 115/ 77) or medical conditions that may had affected fluid balance (i.e. any kind of self-reported liver and kidney condition, *n* = 66/ 79; out of range hematocrit values [<39 and >52 %], *n* = 195/ 223; thyroid, *n* = 141/ 138; metabolic diseases, *n* = 1,954/ 2,368; or currently under any kind of medication, *n* = 198/ 291) were filtered out of the database (Figure NHANES 1). Only participants aged between 19 and 64 years old were retained (*n* = 4,861/ 4,469), with a Body Mass Index (BMI, kg/m^2^) comprised between 17 and 37 (*n* = 4,142/ 3,737) and categorized with dietary recall status as reliable (*n* = 3,949/ 3,391). Additionally, only data from participants with afternoon and evening visits were considered (*n* = 2153/ 886) as only mid- to late- afternoon urine spot samples may accurately estimate 24h urine concentration [3]. Participants with missing urine samples were excluded (*n* = 29/ 18). The resulting dataset was made of 2,992 (*n* = 1,838/ 1,154) rows, where each row represented a different subject, and 81 columns, one for each observed variable. The dataset was then randomly split into training and test sets using a 80:20 ratio.


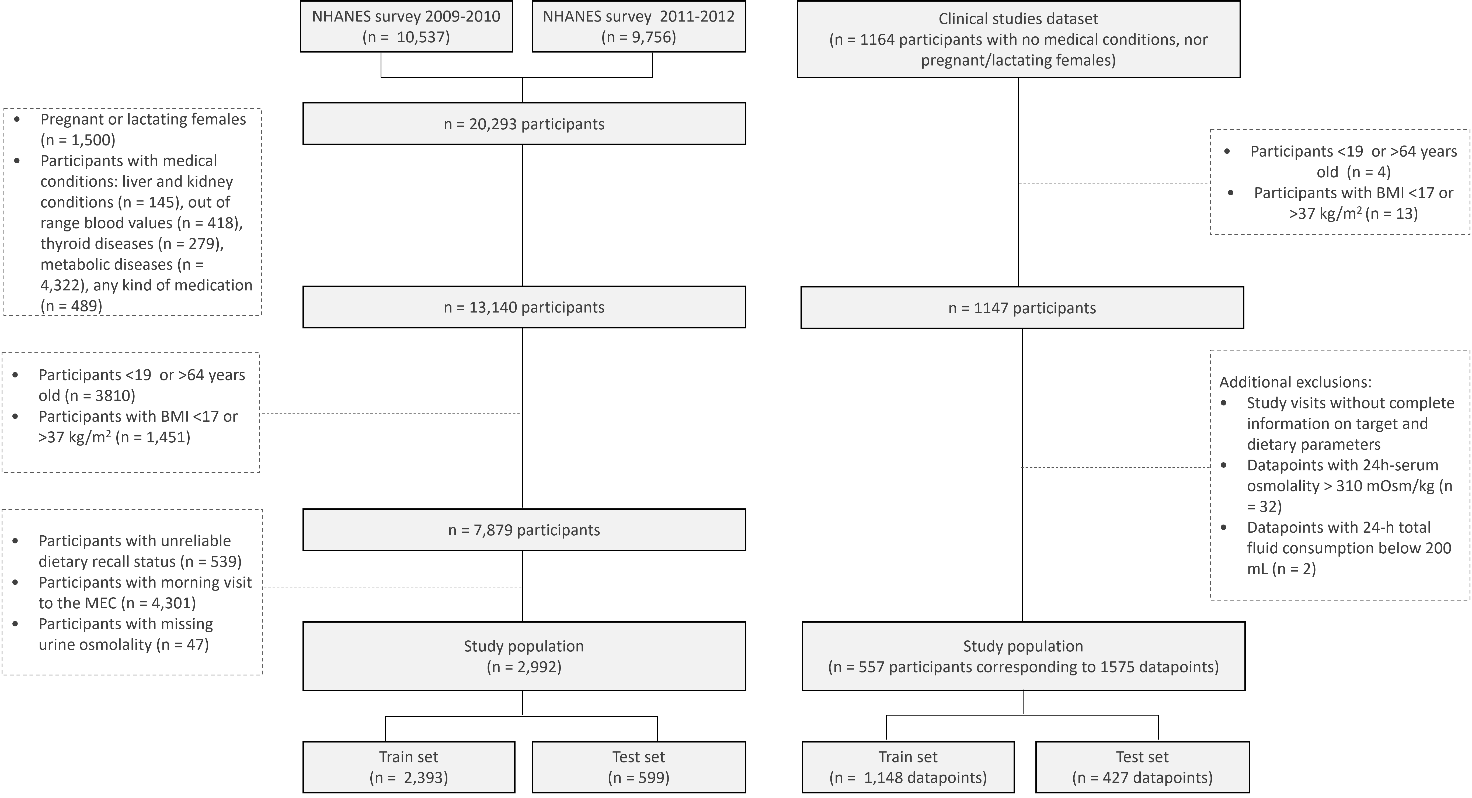


**Figure NHANES 1.** Participant flowchart from the population of adult participants in the NHANES 2009 – 2012.

**Feature selection**

To identify the most relevant variables to predict U_Osm_ in the NHANES dataset, a method derived from Random Forest (RF) methodology, namely Boruta [4], was considered. This algorithm was applied on the whole set of available features. Applying Boruta methodology allowed to attribute importance to all variables examined.

Boruta is an iterative feature selection technique which rely on the assumption that relevant variables should have significantly higher importance than random variables, achieved by shuffling the original ones (i.e. shadow copies), which are reasonably expected to have no importance. In particular, this algorithm at each iteration firstly creates a shadow copy of each available variable, which is shuffled and added to the original dataset. Then a random forest is applied on the new dataset (containing both original variables and shadow copies) and the importance of each feature is measured. The algorithm now assigns a hit to each variable being more important than the most important shadow feature. Considering each individual original variable, it then compares the number of received hits (H) to the expected one (E=b∙0.5, where b is the iteration number) and it deems the variable:

- important if H is significantly higher than E
- unimportant if H is significantly lower than E

All the shadow attributes are finally removed and the previous steps are repeated until all variables have been deemed important or unimportant or the maximum number or iterations has been reached.

Prior to applying this technique to the NHANES dataset, many missing data needed to be addressed. (**Table NHANES 1**). For this reason, imputation of the missing values was applied using k-Nearest Neighbors (kNN) [5] (*n* = 2,992). This implied the assumption of missing at random (MAR), as done in other studies involving the use of data from NHANES [6, 7].

**Table NHANES 1.** % missing data for the main features in NHANES dataset

|  | **A** Variable | Missing values (%) |
| --- | --- | --- |
|  | Age | 0 |
|  | Sex | 0 |
|  | Height | 11.1 |
|  | Weight | 4.37 |
|  | BMI | 11 |
|  | U_Osm_ | 23.19 |
|  |  |  |
|  | **B** Variable | Missing values (%) |
|  | Age (yrs.) | 0 |
|  | Calcium intake (mg) | 8.7 |
|  | Sugar Sweetened Beverages (g) | 33 |
|  | Vegetables (g) | 27 |
|  | Non alcoholic Beverages (g) | 44 |
|  | Serum Osmolality (mmol/Kg) | 7.5 |
|  | Sugars, Sweets, Beverages (g) | 9.1 |
|  | Milk (g) | 30 |
|  | Total sugars (g) | 8.8 |
|  | Fats, oils, salad dressings (g) | 8.8 |

Table NHANES 1: % of missing data for main features in NHANES dataset. Table **A** presents % of missing values for participants demographic characteristics in (before imputation and filtering). Table **B** presents % of missing values for top 10 features to determine U_Osm_ after filtering.

After applying Boruta to remove redundant and irrelevant variables, Extreme Gradient Boosting (XGBoost) was adopted to achieve the preliminary identification of the most relevant features linked to urine production in NHANES dataset (see Table NHANES 2 for hyperparameters values).

**Table NHANES 2. Hyperparameters’ configuration for feature selection**

| **Hyperparameter** | **NHANES** |
| --- | --- |
| ntrees | 565 |
| sample_rate | 0.8 |
| max_depth | 4 |
| min_split_improvement | 1e-6 |
| nbins | 16 |
| nbins_cats | 16 |
| min_rows | 16 |
| col_sampe_rate | 0.8 |
| col_sampe_rate_per_tree | 0.6 |
| learn_rate_annealing | 0.99 |
| learn_rate | 0.1 |

## Results

**Demographic characteristics**

Data are presented as mean (min; max) or as % of the respective dataset, unless specified otherwise.

The average age of the observed individuals was 39 (19; 64) years both in the train and test set in the NHANES dataset (**Figure NHANES 2**). Both train and test sets had a higher percentage of male participants (61% and 55%, respectively); average height was 170 (140; 203) cm for males and 169 (144; 205) cm for women. BMI was 26.8 kg/m^2^ in both sets. U_Osm_ ranged between 45 and 1,294 mOsm/kg in the train set, with a mean value equal to 660 mOsm/kg. A similar mean value was found in the test set with a range of [84 - 1,239] mOsm/kg.

**Figure NHANES 2.** Comparison of train and test set in NHANES dataset.


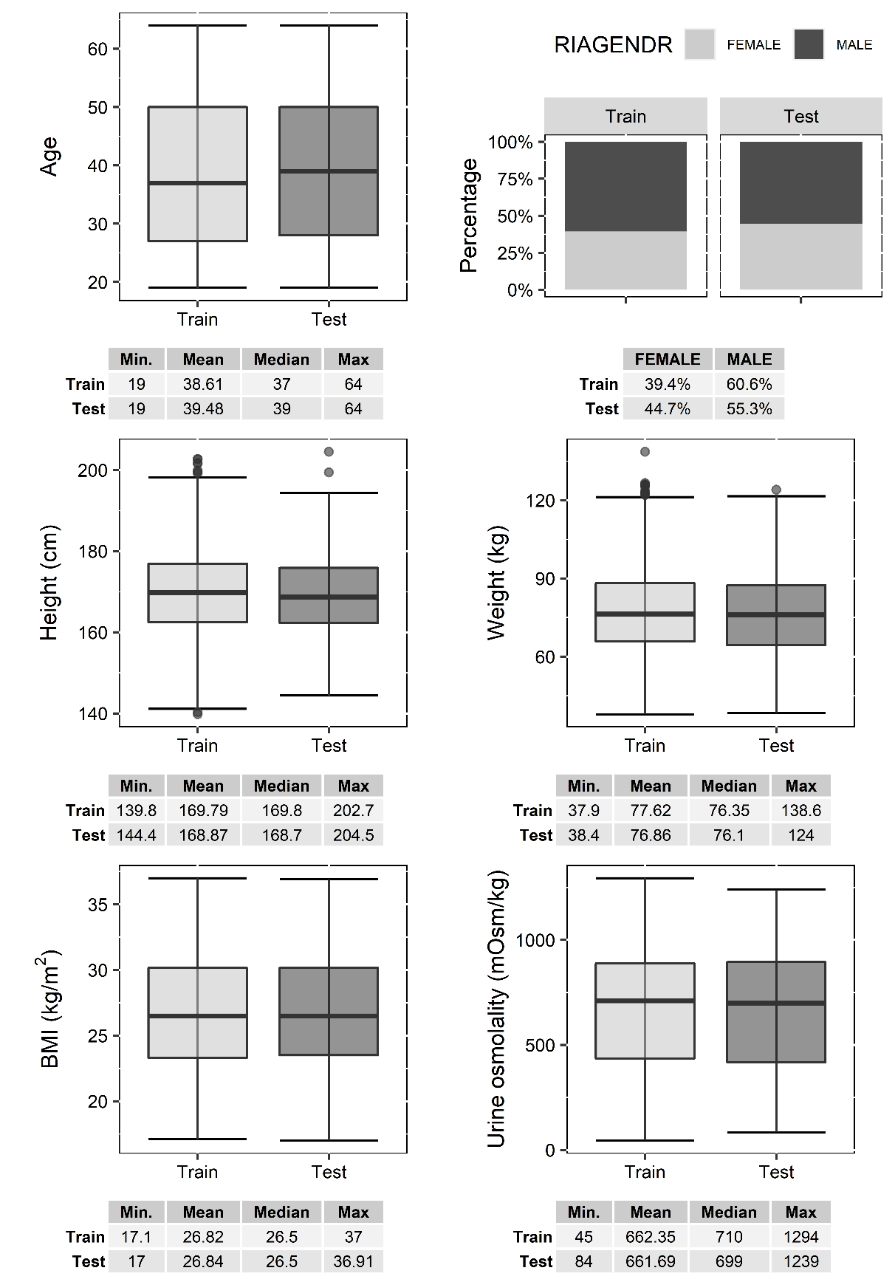


**Feature selection**

The adoption of Xgboost algorithm lead to the selection of 42 relevant variables. The preliminary identification of the most relevant features linked to urine production in NHANES dataset is shown in **Figure NHANES 3.** The features are ranked based on their fractional contribution to the model, i.e. the total gain of each feature's splits (x-axis of Figure NHANES 3 - A). The most predictive feature, Age, showed a low fractional contribution of 0.06. Additionally, no physiological variables (e.g. Urine Volume) nor fluid intake variables known to be linked with urine osmolality ranked among the key contributing features. This showed the inadequacy of data imputation for the current study to achieve a better data quality to the end of improving predictions' precision.


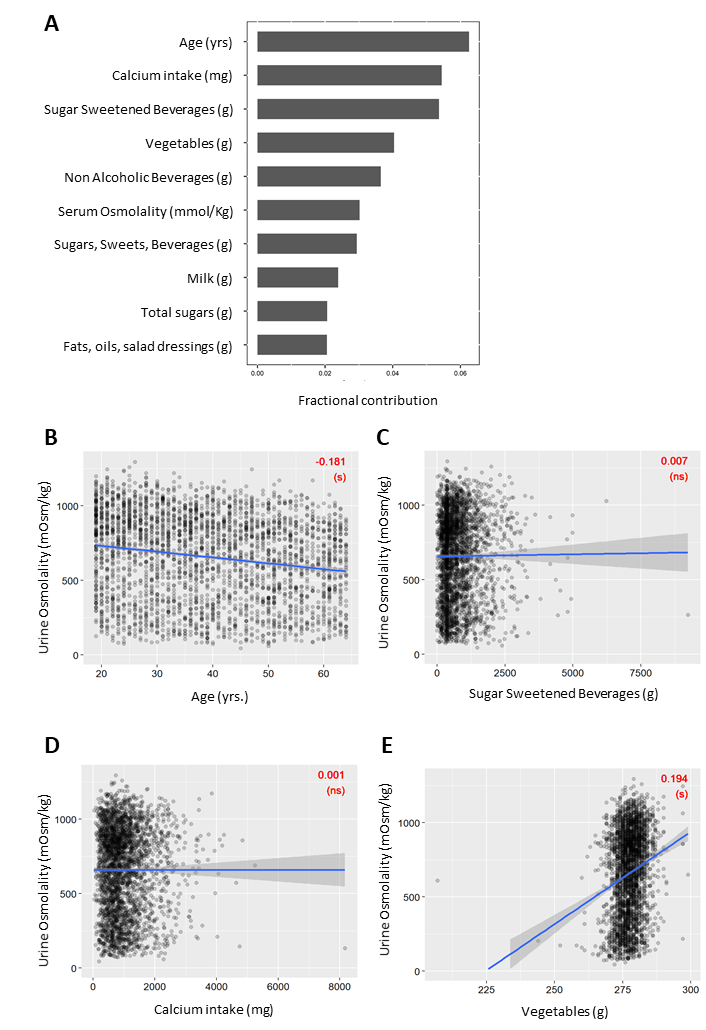


**Figure NHANES 3.** Variable importance to determine U_Osm_ in NHANES dataset and data distribution. (A) Feature importance plot showing the 10 most important features in NHANES raw data obtained with XGBoost. (B-E) Data distribution of Urine Osmolality (U_Osm_) against Age, Sugar-sweetened-beverages (SSB), Calcium intake and Vegetables (*n* = 2,992). Pearson’s correlation coefficient; (s) significant, (ns) non-significant based on linear regression p-value (< 0.05). The R ggplot2 package was used to generate the figures <https://ggplot2.tidyverse.org>. R Statistical Software version 3.6.3 (R Core Team https://cran.r-project.org/bin/windows/base/old/3.6.3/).

Therefore, it was concluded that the NHANES dataset was not suitable to generate predictions of U_Osm_. This implied a complete re-think on the data analysis approach that was used so far. Hence the decision to perform the analysis with a new source of data with more context-specific data deriving from clinical studies aimed to experimentally investigate hydration physiology where quality and completeness of the data was ensured.

# Reference List

1. Friedman, J.H., *Greedy Function Approximation: A Gradient Boosting Machine.* The Annals of Statistics, 2001. **29**(5): p. 1189-1232.

2. Apley, D.W. and J. Zhu, *Visualizing the effects of predictor variables in black box supervised learning models.* Journal of the Royal Statistical Society: Series B (Statistical Methodology), 2020. **82**(4): p. 1059-1086.

3. Bottin, J.H., et al., *Equivalence of afternoon spot and 24-h urinary hydration biomarkers in free-living healthy adults.* Eur J Clin Nutr, 2016. **70**(8): p. 904-7.

4. Kursa, M.B. and W.R. Rudnicki, *Feature Selection with the Boruta Package.* 2010, 2010. **36**(11): p. 13.

5. Kowarik, A. and M. Templ, *Imputation with the R Package VIM.* Journal of Statistical Software, 2016. **074**(i07).

6. Wang, Y.C., et al., *Prevalence of Masked Hypertension Among US Adults With Nonelevated Clinic Blood Pressure.* Am J Epidemiol, 2017. **185**(3): p. 194-202.

7. Barnard, J. and X.L. Meng, *Applications of multiple imputation in medical studies: from AIDS to NHANES.* Stat Methods Med Res, 1999. **8**(1): p. 17-36.
